# LINC00667 Sponges miR-4319 to Promote the Development of Nasopharyngeal Carcinoma by Increasing FOXQ1 Expression

**DOI:** 10.3389/fonc.2020.632813

**Published:** 2021-01-25

**Authors:** Bing Liao, Yun Yi, Lei Zeng, Zhi Wang, Xinhua Zhu, Jianguo Liu, Bingbin Xie, Yuehui Liu

**Affiliations:** ^1^Department of Otorhinolaryngology Head and Neck Surgery, The Second Affiliated Hospital of Nanchang University, Nanchang, China; ^2^Department of Gynaecological Oncology, Jiangxi Cancer Hospital, Nanchang, China; ^3^Department of Oncology, The Second Affiliated Hospital of Nanchang University, Nanchang, China

**Keywords:** nasopharyngeal carcinoma, LINC00667, miR-4319, FOXQ1, cancer development

## Abstract

Accumulating evidence has indicated that lncRNAs regulate various biological and pathological processes in diverse malignant tumors. The roles of LINC00667 in cancer development have been explored in glioma, hepatocellular carcinoma and non-small cell lung cancer, but not in nasopharyngeal carcinoma (NPC). In the present study, we characterize the role and molecular mechanism of LINC00667 in NPC progression. It was found that LINC00667 was overexpressed in NPC cells compared to normal cells. Silencing LINC00667 suppressed the proliferation, migration, invasion and epithelial mesenchymal transition (EMT) in NPC cells. In addition, bioinformatics analysis revealed that LINC00667 acted as a ceRNA to absorb miR-4319. Further investigations illustrated that miR-4319 had low expression in NPC cells and functioned as a tumor suppressor in the progression of NPC. Mechanistic study identified forkhead box Q1 (FOXQ1) as a functional target of miR-4319. The effect of LINC00667 in NPC development was mediated by the miR-4319/FOXQ1 axis. Analysis on tumorxenograft mouse model demonstrated that knockdown of LINC00667 repressed NPC tumor growth *in vivo* and confirmed the *in vitro* results. Our present study suggested that LINC00667 promoted the malignant phenotypes of NPC cells by competitively binding to miR-4319 to up-regulate FOXQ1 expression. Our results reveled that LINC00667 could be a diagnostic and therapeutic target for NPC patients.

## Introduction

Nasopharyngeal carcinoma (NPC) is one of the most frequent malignant head and neck tumors, which originate from in the surface epithelium of nasopharynx (1, 2). The prevalence of NPC is geographically concentrated in Southeast Asia, especially in Southern China (3). Currently, the most commonly used therapeutic strategies for NPC are chemotherapy and radiation therapy, but the low sensitivity to chemotherapeutic drugs as well as the development of multidrug resistance hinder the treatment efficacy, leading to high recurrence rate and poor outcome of NPC (4–6). The pathogenesis of NPC is complex and involves in a variety of factors including diet, genetic susceptibility, viral infection and carcinogens (7). Thus, exploring the pathological mechanism governing NPC tumorigenesis is urgently needed for the treatment of NPC.

It is well known that long noncoding RNAs (lncRNAs) is a class of ubiquitous transcript greater than 200 nucleotides without protein coding ability (8, 9). Dysregulated lncRNAs have been detected in a wide range of pathological states, including human cancers (10, 11). Accumulating evidence has illustrated that lncRNAs are implicated in the initiation and progression of NPC. For example, up-regulated lncRNA AFAP1-AS1 expression is associated with NPC progression and poor prognosis (12). LncRNA LET repressed by EZH2 suppresses NPC cell proliferation and induces apoptosis (13). The p53-regulated lncRNA LOC401317 restrains cell proliferation and promotes apoptosis in NPC (14). Hence, it is important to identify the expression patterns and functional roles of lncRNAs in the tumorigenesis and development of NPC.

LncRNALINC00667 is located in human chr18 (5238100–5246508) with a full length of 8,409 bps and contains multiple miRNA response element (MRE) regions (15, 16). A number of studies have demonstrated that LINC00667 functions as a key mediator in the processes of diverse malignancies. For instance, LINC00667 facilitates the formation of glioma vasculogenic mimicry and the level of LINC00667 is positively associated with the development of glioma (17). LINC00667 predicts the poor prognosis of hepatocellular carcinoma (18). Moreover, overexpression of LINC00667 is strongly correlated with overall survival of patients with non-small cell lung cancer (NSCLC) (19). However, the role of LINC00667 in NPC and its mechanism have not been clarified. In our study, we explore the expression of lncRNA LINC00667 in NPC and how it affects the malignant phenotypes of NPC cells.

## Methods

### Cell Culture

Human NPC cell lines (SUNE-1, CNE-1, CNE-2, HONE-1, HNE-1) and human normal nasopharyngeal epithelium cell line NP69 were purchased from the Chinese Academy of Sciences (Shanghai, China). All cell lines were maintained in DMEM/RPMI 1640 medium supplemented with 10% fetal bovine serum and cultured in a humidified incubator containing 5% CO_2_ at 37°C.

### Gene Transfection

The LINC00667-specific shRNA (sh-LINC00667) and the FOXQ1-specific shRNA (sh-FOXQ1) were used to knock down LINC00667 and FOXQ1, respectively. Non-specific sh-RNA (sh-NC) was used as a negative control. The miR-4319 mimics, inhibitors and the negative controls (NC mimic and NC inhibitor) were synthesized by Invitrogen (Carlsbad, USA). The transfections were carried out by Lipofectamine 3000 reagent as recommended by the vender.

### Reverse Transcription-Quantitative Polymerase Chain Reaction

RT-qPCR was performed to detect the gene expression level. Firstly, the TRIzol reagent was used to extract the total RNA from NPC cells. And then RNA purity was measured by Nano Drop 2000 spectrometer. After that, under the manufacturer’s protocols, cDNA was synthesized with the Reverse Transcription System Kit. The PCR reaction was carried out using SYBR qPCR Mix (Toyobo, Osaka, Japan) and results were calculated using 2^-ΔΔCt^ method. GAPDH and U6 served as internal controls for normalization.

### Cell Proliferation Assays

Cell viability was assessed using Cell Counting Kit-8 (MedChem Express, Monmouth Junction, USA) according to the manufacturer’s protocol. For 5-Ethynyl-20-deoxyuridine (EdU) assay, NPC cells were seeded onto 24-well tissue culture plates, and then detected by the EdU assay kit (Ribo, Guangzhou, China) according to the product manual. The number of EdU-positive cells was counted under a microscope (Olympus, Tokyo, Japan) in three random fields using a 100 × objective (Olympus, Tokyo, Japan).

### Cell Migration and Invasion Assays

The wound healing and transwell chamber assays were performed to examine cell migration and invasion ability. The NPC cells were cultured in six-well plate until >90% confluence. Wounds were created *via* a scratch using pipette tips in the wound healing assay. Besides, NPC cells were cultured in serum-free medium at 37°C for 48 h. The wound healing results were observed under an inverted microscope at 0 and 48 h after cells were scratched.

For transwell chamber assays, transwell chambers (Corning, Cambridge, MA) covered with or without matrigel (BD Biosciences, CA, USA) were used for assessing cell migration and invasion ability. A total of approximately 1 × 10^6^ cells was added into the top chamber of 24-well plates, and then the bottom well was complemented with 20% FBS. After incubating for 24 h, the migrated or invaded cells were fixed with 4% paraformaldehyde and then stained with Crystal violet. After that, cells were counted and imaged under an inverted microscope (Olympus, Tokyo, Japan).

### Western Blot Analysis

Cell proteins lysates were separated by 10% SDS-PAGE and transferred to nitrocellulose (NC) membranes. The membranes were blocked with 5% non-fat milk for 1 h at room temperature. Specific primary antibodies against E-cadherin (ab231303, 1:1,000, abcam) and Vimentin(ab20346, 1: 1,000, abcam)were added for overnight incubation at 4°C, followed by incubation with HRP-labeled secondary antibodies and Enhanced Chemiluminescence (ECL) Kit (Thermo Scientific, CA, USA) was used to measure the immunoreactivity.

### Subcellular Fractionation

Cytoplasmic and Nuclear RNA Purification Kit (Norgen, Belmont, CA, USA) was used for detection of lncRNA LINC00667 nucleus fraction and cytoplasmic fraction isolated from NPC cells.

### RNA Immunoprecipitation Assay

RNA-Binding Protein Immunoprecipitation Kit (Millipore, Bedford, MA) was applied to conduct RIP experiment. Immunoprecipitation was carried out with Ago2 and IgG utilized as the negative controls. The abundance of LINC00667 and miR-4319 in precipitated RNAs was determined by RT-qPCR analysis.

### Luciferase Reporter Assay

The segments of LINC00667 and FOXQ1 containing miR-4319 binding sites were ligated into pmirGLO vectors. The mutant plasmids were constructed by mutating binding sites with miR-4319. SUNE-1 and HNE-1 cells were co-transfected with indicated reporter vectors and miR-4319 mimics or NC mimics utilizing Lipofectamine 3000 following the manufacture’s procedures. The luciferase activity was measured after transfection.

### RNA Pull-Down Assay

RNA pull-down assay was performed with the Pierce™ Magnetic RNA-Protein Pull-Down Kit (#20164, Thermo Fisher Scientific) in line with the manufacturer’s recommendations.

### *In Vivo* Tumor Growth

All animal procedures were approved by the Animal Care Committee of The Second Affiliated Hospital of Nanchang University. BALB/c nude mice were purchased from Vital River Laboratory. 5 × 10^6^ HNE-1 cells stably transfected with sh-LINC00667 or sh-NC were subcutaneously injected into the nude mice. Tumor volume was measured every 7 days and calculated by the following formula: volume (mm^3^) = (length ×width^2^)/2. By the end of experiments, mice were euthanized. Tumor tissues from all groups were dissected and weighted. The experimental protocol was established according to the ethical guidelines of the Helsinki Declaration and was approved by the Animal Ethics Committee of the Second Affiliated Hospital of Nanchang University.

### Hematoxylin and Eosin Staining Assay

Tumors from nude mice were fixed with 10% buffered formalin at room temperature for 48 h, then dehydrated and embedded in paraffin. The sections were dyed with H&E solution and photographed using a light microscope.

### Immunohistochemistry Assay

Paraffin-embedded tissue sections were deparaffinized in xylene, dehydrated with ethanol, and blocked by 3% H_2_O_2_ for 10 min. Following antigen retrieval in EDTA buffer at 95°C for 15–20 min, sections were incubated with Ki67 antibody (ab92742, 1: 1,000, abcam) overnight at 4°C. After washing three times with PBS, the secondary antibody coupled to HRP was incubated at 37°C for 50 min. Then, DAB chromogen was added, sections were washed with water and counterstained with hematoxylin.

### TUNEL Staining

Using a TUNEL assay kit (Roche, USA) to detect the apoptosis of tumor sections. The sections were washed by PBS and then immobilized for 30 min with 4% paraformaldehyde. After washed with PBS once, the slices were added with 0.1% Triton X-100 for 2 min, and then washed with PBS once. Afterwards, 3% H_2_O_2_ was used for incubation (5 min). Then the slides incubated with terminal deoxynucleotidyl transferase (TdT) enzyme at 37°C overnight. Then, sections were incubated with antidigoxigenin-peroxidase conjugate and using DAB to evaluate activity. The sections were examined under a light microscope and the TUNEL-positive cells were calculated in Image J software.

### Statistical Analysis

SPSS (18.0 version, SPSS, Inc., USA) and GraphPad (6.0 version) were used to analyze statistical data. Data were represented as mean ± SD. Moreover, student’s t-test and one-way ANOVA were used for comparison between two or more groups. All the experiments were repeated at least three times. *P*< 0.05 was considered statistically significant.

## Results

### Down-Regulation of LINC00667 Impedes NPC Cell Proliferation

**Figure 1:**
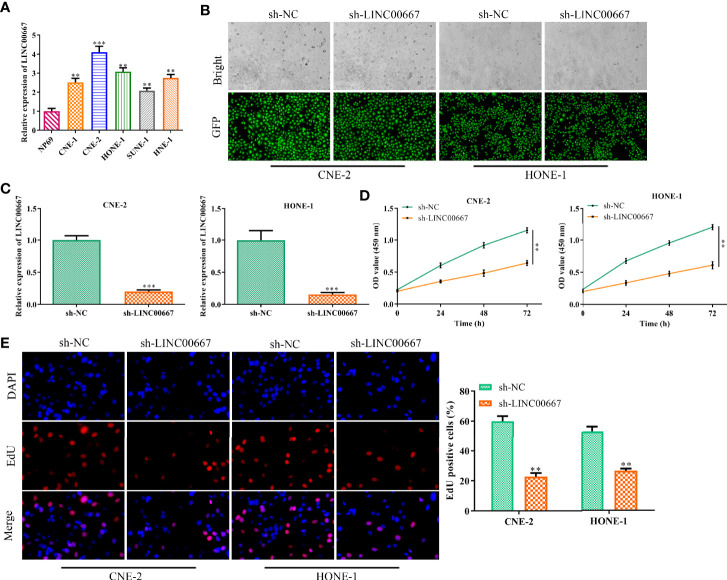
Downregulation of LINC00667 impedes nasopharyngeal carcinoma (NPC) cell proliferation. **(A)** Reverse transcription-quantitative polymerase chain reaction (RT-qPCR) analysis of long noncoding RNA (lncRNA) LINC00667 level in human NPC cell lines (SUNE-1, CNE-1, CNE-2, HONE-1, and HNE-1) as well as human normal nasopharyngeal epithelium cell line NP69. **(B)** GFP assay and **(C)** RT-qPCR analysis on the gene transfection efficiency. **(D)** CCK-8 and **(E)** 5-Ethynyl-20-deoxyuridine (EdU) assay detected the impacts of LINC00667 on the proliferative capacity of NPC cells transfected with sh-LINC00667. Magnification 200×. All experiments were performed at least in triplicate. ^**^P < 0.01, ^***^P < 0.001 *vs.* NP69 group or sh-NC group.

To determine the abundance of LINC00667 in NPC cells, RT-qPCR analysis was carried out. We observed that the level of LINC00667 in NPC cell lines (CNE-1, CNE-2, HONE-1, SUNE-1 and HNE-1) was markedly higher than that in normal cell line NP69 (**Figure 1A**). As a result, we stably down-regulated LINC00667 expression by shRNA was used to explore the biological role of LINC00667 in NPC development. As shown in **Figures 1B, C**, the efficiency of LINC00667 interference was certified by GFP and RT-qPCR assays. Then, CCK8 assay showed that inhibition of LINC00667 resulted in the decrease of NPC cell viability (**Figure 1D**). Likewise, EdU assay indicated that down-regulation of LINC00667 dramatically inhibited NPC cell proliferation (**Figure 1E**). These results demonstrated that LINC00667 was up-regulated in NPC and promoted cell proliferation of NPC.

### Down-Regulation of LINC00667 Suppresses Nasopharyngeal Carcinoma Cell Migration, Invasion and EMT

**Figure 2:**
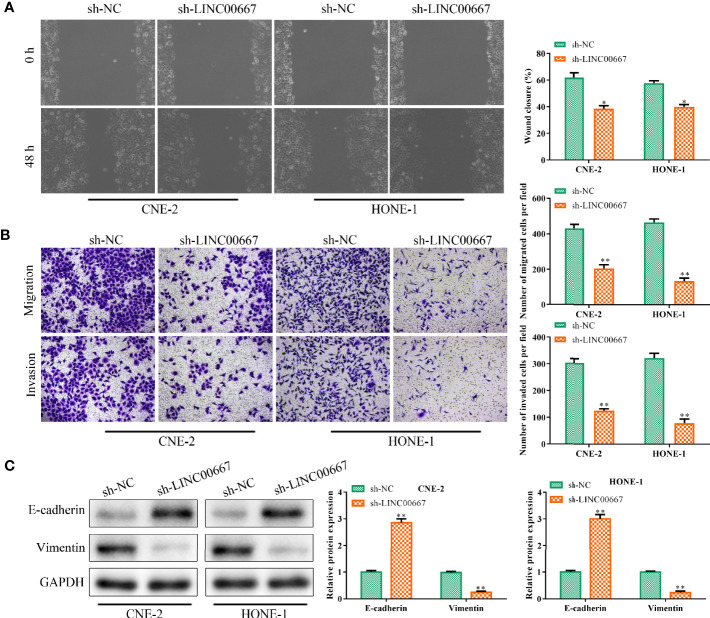
Downregulation of LINC00667 suppresses nasopharyngeal carcinoma (NPC) cell migration, invasion and epithelial mesenchymal transition (EMT). **(A)** Wound-healing assay and **(B)** transwell chamber assay to measure the migration and invasion abilities of NPC cells transfected with sh-LINC00667. **(C)** The protein expression levels of EMT biomarkers. All experiments were performed at least in triplicate.^*^P < 0.05, ^**^P < 0.01 *vs.*sh-NC group.

Based on the above findings, we further explore the function of LINC00667 in cell migration and invasion. Wound-healing assay illuminated that LINC00667 silencing led to the suppression of cell migration (**Figure 2A**). Similarly, results of transwell chamber assay suggested that the invasion ability of NPC cells was inhibited by attenuation of LINC00667 (**Figure 2B**). For the reason that EMT plays a vital part in cell metastasis, Western blot was used to measure the expression levels of EMT biomarkers so as to identify the effects of LINC00667 on EMT. Results delineated that knockdown of LINC00667 increased the expression of epithelial biomarker E-cadherin and declined the expression level of the mesenchymal biomarker (Vimentin) in SUNE-1 and HNE-1 cells (**Figure 2C**). These results indicated that LINC00667 down-regulation inhibited the migration, invasion and EMT of NPC cells.

### LINC00667 Acts as a Molecular Sponge for miR-4319

**Figure 3:**
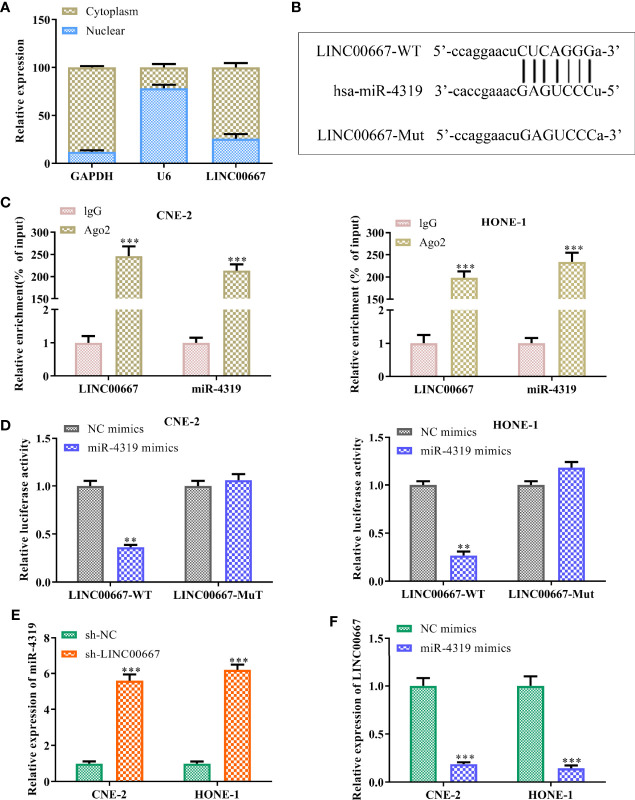
LINC00667 acts as a molecular sponge for miR-4319. **(A)** The subcellular localization of LINC00667. **(B)** Bioinformatics analysis on the putative binding sites of LINC00667 in miR-4319. **(C)** RNA immunoprecipitation (RIP) assay. **(D)** Luciferase reporter assay. **(E, F)** RT-qPCR analysis of LINC00667 and miR-4319 expression levels in NPC cells transfected with miR-4319 mimic or sh-LINC00667. Magnification 200×. All experiments were performed at least in triplicate.^**^P < 0.01, ^***^P < 0.001 *vs.* IgG group/NC mimic group/sh-NC group.

To further understand the mechanism of LINC00667 in promoting NPC cell growth, we detected the subcellular localization of LINC00667 and found that LINC00667 was markedly distributed in the cytoplasm of SUNE-1 and HNE-1 cells (**Figure 3A**). Accordingly, we conjectured that LINC00667 exerted its function *via* a competitive endogenous RNA (ceRNA) pattern. By exploring Starbase website, we uncovered that miR-4319 owned the putative binding sites for LINC00667 (**Figure 3B**). Therefore, RIP and luciferase reporter assays were carried out to confirm the interaction between LINC00667 and miR-4319. Results showed that LINC00667 and miR-4319 were highly enriched by Ago2 relative to IgG (**Figure 3C**). In concert with these results, miR-4319 mimics suppressed the luciferase activity of LINC00667-WT reporter in NPC cells, whereas efficacy was lost in response to LINC00667-Mut (**Figure 3D**). Moreover, we observed that miR-4319 expression was significantly higher in cells transfected withsh-LINC00667 than that in sh-NC group (**Figure 3E**). LINC00667 was weakly expressed when miR-4319 was up-regulated (**Figure 3F**). To sum up, these findings suggested that miR-4319 was sponged by LINC00667.

### MiR-4319 Inhibits the Malignant Behaviors of Nasopharyngeal Carcinoma Cells

**Figure 4:**
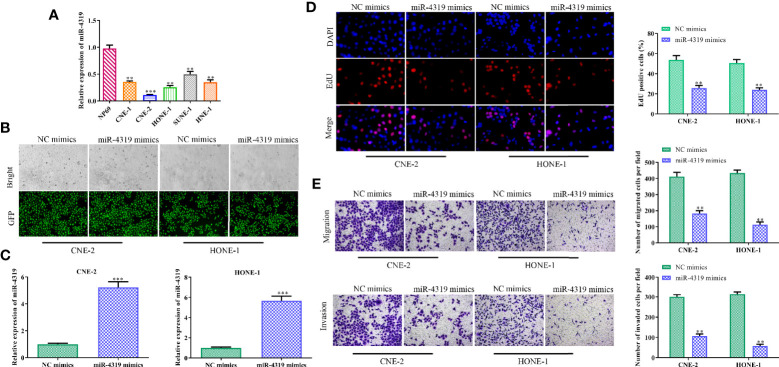
MiR-4319 inhibits the malignant behaviors of nasopharyngeal carcinoma (NPC) cells. **(A)** Reverse transcription-quantitative polymerase chain reaction (RT-qPCR) on the expression pattern of miR-4319 in NPC cells. **(B, C)** Determination of the efficiency of miR-4319 overexpression by GFP and RT-qPCR assays. **(D)** EdU assay. **(E)** Transwell chamber assay. Magnification 200×. All experiments were performed at least in triplicate. ^**^P < 0.01, ^***^P < 0.001*vs.* NP69 group or NC mimic group.

Although miR-4319 has been testified to be a tumor suppressor in various cancers, the role of miR-4319 on NPC cells is not clear. Thus, a series of experiments were conducted to characterize the role of miR-4319 in NPC. In comparison with normal cells, miR-4319 was down-regulated in NPC cells (**Figure 4A**). Because SUNE-1 and HNE-1 cells presented the lowest expression levels of miR-4319 among the five NPC cell lines, they were selected for transfection with miR-4319 mimics. GFP and RT-qPCR confirmed that miR-4319 was overexpressed in SUNE-1 and HNE-1 cells after transfection (**Figures 4B, C**). EdU assay showed that overexpression of miR-4319 significantly inhibited NPC cell proliferation (**Figure 4D**). Moreover, transwell chamber assay indicated that enhanced expression of miR-4319 overtly suppressed the migration and invasion of SUNE-1 and HNE-1 cells, (**Figure 4E**). These results suggested that miR-4319 played an inhibitory role in NPC progression.

### LINC00667 Elevates FOXQ1 Level in a miR-4319-Mediated Mechanism

**Figure 5:**
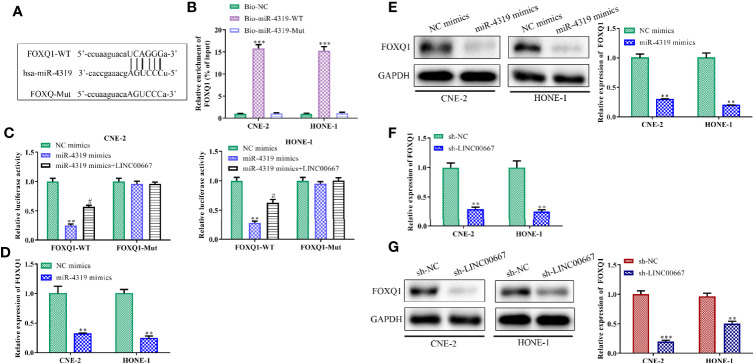
LINC00667 elevates FOXQ1 level in a miR-4319-mediated mechanism. **(A)** The predicted binding regions between FOXQ1 and miR-4319. **(B)** RNA pull-down assay. **(C)** Luciferase reporter assay. **(D–G)** Reverse transcription-quantitative polymerase chain reaction (RT-qPCR) and Western blot analyses of FOXQ1 level in nasopharyngeal carcinoma (NPC) cells transfected with miR-4319 mimic or sh-LINC00667. All experiments were performed at least in triplicate. ^**^P < 0.01, ^***^P < 0.001 *vs.* Bio-NC group/NC mimic group/sh-NC group. ^#^*P* < 0.05 *vs.*miR-4319 mimic group.

Bioinformatics analysis was used to search the downstream genes of miR-4319. It was predicted that FOXQ1 might be a direct target of miR-4319 (**Figure 5A**). To validate the association among FOXQ1, miR-4319 and LINC00667, RNA pull-down assay, luciferase reporter assay, RT-qPCR and Western blot were performed. Our observations illustrated that FOXQ1 was abundantly expressed in compounds pulled down by biotin-labeled miR-4319-WT (**Figure 5B**). Consistently, miR-4319 mimics remarkably impaired the luciferase activity of FOXQ1-WT and overexpression of LINC00667 led to the recovery of FOXQ1-WT activity, while the mutant forms of FOXQ1 had no notable changes (**Figure 5C**). Results of RT-qPCR and Western blot delineated that the expression level of FOXQ1 was lessened by miR-4319 mimics or depletion of LINC00667 (**Figures 5D–G**). Collectively, the described findings disclosed that LINC00667 up-regulated FOXQ1 through competing for miR-4319.

### LINC00667 Promotes Nasopharyngeal Carcinoma Progression by Regulating miR-4319/FOXQ1 Axis

**Figure 6:**
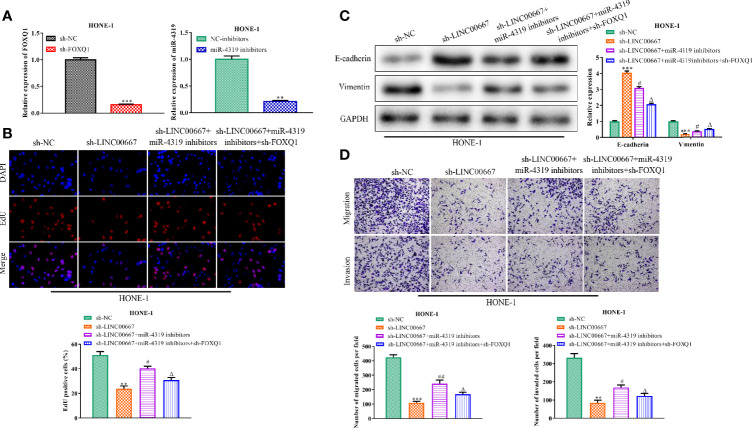
LINC00667 promotes nasopharyngeal carcinoma (NPC) progression by regulation of miR-4319/FOXQ1 axis. **(A)** Expression levels of miR-4319 and FOXQ1 detected by reverse transcription-quantitative polymerase chain reaction (RT-qPCR) assay. **(B)** 5-Ethynyl-20-deoxyuridine (EdU) assay assessed the proliferative capacity of NPC cells. **(C)** Western blot analysis of protein levels of EMT-related biomarkers. **(D)** Detection of cell migration and invasion by transwell chamber assay. Magnification 200×. All experiments were performed at least in triplicate. ^**^P < 0.01, ^***^P < 0.001 *vs.* sh-NC group. ^#^*P* < 0.05, ^##^*P* < 0.01 *vs.* sh-LINC00667 group. ^△^*P* < 0.05 *vs.* sh-LINC00667+miR-4319 inhibitor group.

In order to further identify whether miR-4319 and FOXQ1 were involved in LINC00667-mediated promotion of the proliferation, migration, invasion and EMT in NPC cells, rescue experiments were carried out. RT-qPCR analysis demonstrated that miR-4319 expression was inhibited after transfection with miR-4319 inhibitor and FOXQ1 was silenced after transfection with sh-FOXQ1 (**Figure 6A**). EdU assay revealed that the proliferation of LINC00667-downregulated cells was increased by suppression of miR-4319 and retrieved by knockdown of FOXQ1 (**Figure 6B**). Furthermore, miR-4319 inhibitors led to the diminution of E-cadherin level and the increase in the expression level of Vimentin in HNE-1 cells transfected with sh-LINC00667, meanwhile, FOXQ1 silence abolished the impacts of miR-4319 inhibitors on the expression levels of E-cadherin and Vimentin (**Figure 6C**). In agreement with the foregoing findings, transwell chamber assays illuminated that cell migration and invasion suppressed by LINC00667 knockdown were facilitated by miR-4319 inhibitors and then reversed by down-regulation of FOXQ1 (**Figure 6D**). In a word, the miR-4319/FOXQ1 pathway mediated the function of LINC00667 in NPC.

### LINC00667 Knockdown Inhibits Tumor Growth of Nasopharyngeal Carcinoma *In Vivo*

**Figure 7:**
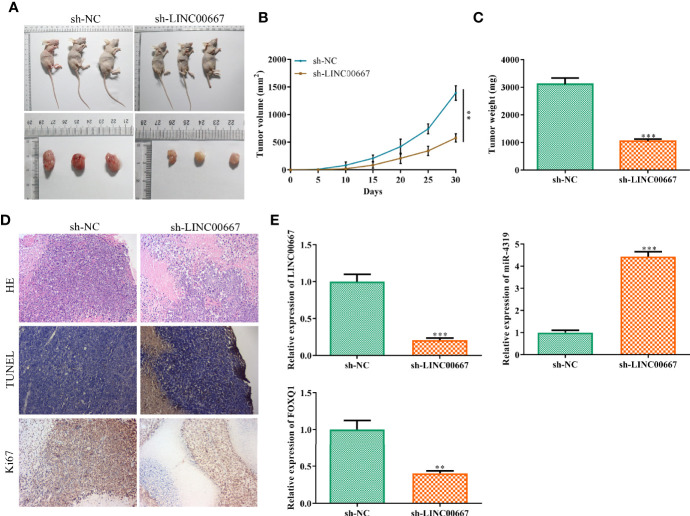
LINC00667 knockdown inhibits tumor growth of nasopharyngeal carcinoma (NPC) *in vivo*. **(A)** Macroscopic images of transplanted tumors. **(B, C)** The volume and weight of xenograft tumors. **(D)** Hematoxylin and eosin (H&E) staining, immunohistochemistry (IHC) of Ki-67 and TUNEL in tumor sections. **(E)** reverse transcription-quantitative polymerase chain reaction (RT-qPCR) on the expression levels ofLINC00667, miR-4319, and FOXQ1 in tumor tissues. All experiments were performed at least in triplicate. ^**^P < 0.01, ^***^P < 0.001 *vs.* sh-NC group.

On the basis of the above findings, we further investigate the impacts of LINC00667 on tumor progression *in vivo*. As shown in **Figures 7A–C**, the volume and weight of subcutaneous tumors were smaller in mice injected with LINC00667-downregulated cells than those in sh-NC group. In addition, immunohistochemical staining and TUNEL staining showed that the proportion of Ki67 positive cells was reduced and cell apoptosis was increased in the LINC00667 down-regulation group (**Figure 7D**). To further verify the relationship between LINC00667, miR-4319 and FOXQ1 *in vivo*, we detected their expression levels in xenografts. Results showed that LINC00667 and FOXQ1 were down-regulated, whereas miR-4319 level was enhanced in tumor tissues from sh-LINC00667 group compared with those from sh-NC group (**Figure 7E**). These results suggested that inhibition of LINC00667 impeded NPC cell growth *in vivo*.

## Discussion

A growing number of evidence affirms that lncRNAs play core roles in a wide range of biological and pathological processes (20), and are involved in the initiation and progression of various types of cancers (10, 11). LncRNALINC00667 has been reported to be a key mediator in the development of glioma (17), hepatocellular carcinoma (18), non-small cell lung cancer (19) and breast cancer (21). In this study, we characterize the expression status of LINC00667 and unravel its molecular mechanism in promoting the development of NPC. Our results revealed that the expression of LINC00667 was prominently elevated in NPC cells compared with normal cells. Loss-of-function assay by knocking downLINC00667 led to the inhibition of NPC cell proliferation, migration, invasion and EMT. These results are in consistent with previous reports that showed aberrant expression levels of lncRNAs are widely observed in numerous malignancies, including NPC (22–24). In addition, a variety of lncRNAs has been reported to be oncogenes or tumor suppressors in the initiation and evolution of NPC. For instance, overexpression of SOX2-activated lncRNA ANRIL promotes NPC cell growth (25). LncRNA THOR attenuates sensitivity of NPC cells to cisplatin by enhancing cell stemness (26). NKILA restrains NPC carcinogenesis and metastasis *via* suppression of NF-κB pathway (27).

It has been demonstrated that lncRNAs modulate tumorigenesis through diverse molecular mechanisms. Accumulating evidence indicates that competing endogenous RNA (ceRNA) regulates the carcinogenesis of cancers, including NPC (28, 29). A myriad of literatures suggests that lncRNAs can execute their functions in NPC *via* acting as ceRNAs (30, 31). In view of the primary distribution of LINC00667 in the cytoplasm, we speculated that LINC00667 may involve in the tumorigenesis of NPC by working as a ceRNA. Our bioinformatics analysis revealed that LINC00667 has the potential miR-4319 binding sites, indicating that it could be a ceRNA of miR-4319. It has been reported that miR-4319 is a tumor suppressor in multiple cancers, such as esophageal squamous cell carcinoma (ESCC) (32), non-small cell lung cancer (33), thyroid cancer (34), colorectal cancer (35) and prostate cancer (36). Results of our RIP and luciferase reporter assays confirmed that LINC00667 serves as a molecular sponge of miR-4319. Besides, NPC cells have low expression of miR-4319 and overexpression of miR-4319 retarded the proliferation, invasion and metastasis of NPC cells, suggesting that miR-4319 could inhibit the malignant behavior of NPC cells.

Aberrant expression ofFOXQ1 has been justified to be associated with the development of diverse cancers (37, 38). More importantly, it is disclosed that the expression of FOXQ1 is significantly elevated in NPC tissues and cells and inhibition of FOXQ1 hindered cell growth, migration and invasion in NPC (39, 40). Among all of the predicted target genes ofmiR-4319, we identified FOXQ1 as a direct target of miR-4319. Our results of RNA pull-down assay, luciferase reporter assay, RT-qPCR and Western blot analysis suggested that the expression of FOXQ1 was inhibited by miR-4319 mimics or depletion of LINC00667, implicating that LINC00667 might upregulate FOXQ1 by inhibiting miR-4319.Moreover, our xenograft mouse model validated the role of LINC0066 in promoting NPC cell growth *in vivo*, and the relationship of LINC0066, miR-4319 and FOXQ1.Our results suggest thatLINC00667 might serve as an oncogene in the development of NPC *via* targeting miR-4319/FOXQ1 axis.

## Conclusions

In summary, our study unveiled that lncRNA LINC00667 exerted an oncogenic function in the development and progression of NPC both *in vitro* and *in vivo*. LINC00667 contributed to NPC tumorigenesis as a ceRNA by impairing miR-4319-dependant FOXQ1 down-regulation. Our findings will enhance the understanding of NPC pathogenesis and facilitate the improvement of lncRNA-guided diagnosis and treatment.

## Data Availability Statement

The original contributions presented in the study are included in the article/supplementary materials; further inquiries can be directed to the corresponding author.

## Ethics Statement

The animal study was reviewed and approved by the Animal Care Committee of The Second Affiliated Hospital of Nanchang University.

## Author Contributions

BL, ZW, and BX conceived and designed the study. YY, XZ, and YL performed the literature search and data extraction. LZ and JL drafted the manuscript. All authors contributed to the article and approved the submitted version.

## Conflict of Interest

The authors declare that the research was conducted in the absence of any commercial or financial relationships that could be construed as a potential conflict of interest.

## Abbreviations

EdU, 5-Ethynyl-20-deoxyuridine; EMT, mesenchymal transition; FOXQ1, fork head box Q1; NPC, nasopharyngeal carcinoma; RIP, RNA immunoprecipitation; RT-qPCR, reverse transcription-quantitative polymerase chain reaction.
